# The Association of Interpersonal Relationships and Social Services with the Self-Rated Health of Spanish Homelessness

**DOI:** 10.3390/ijerph18179392

**Published:** 2021-09-06

**Authors:** Fernando Fajardo-Bullón, Jesús Pérez-Mayo, Igor Esnaola

**Affiliations:** 1 Department of Psychology, Faculty of Education and Psychology, University of Extremadura, Avenida de Elvas s/n, 06006 Badajoz, Spain; 2 Department of Economics, University of Extremadura, 06006 Badajoz, Spain; jperez@unex.es; 3 Department of Development and Educational Psychology, Faculty of Education, University of the Basque Country, UPV/EHU, Avenida de Tolosa, 70, San Sebastián, 20018 Leioa, Spain

**Keywords:** self-rated health, homelessness, interpersonal relationship, social services, Spain

## Abstract

Understanding the specific factors associated with poor health is critical to improve the health of homeless people. This study aimed to analyze the influence of personal variables, interpersonal relationships, and the influence of social services on the health of homeless people. A secondary analysis was applied to cross-sectional data from a sample of 1382 homeless people living in the Basque Country (Spain) (75.69% male). Multinomial logistic regression modelling was used to analyze the relationship between health and personal variables, interpersonal variables, perceived help and use of the social services. Relationships with the family, using a day center, and a sufficient and high perceived help of the social services were significant factors associated with good health. On the other hand, spending the day alone or using mental and health care services are associated with poor health. In the same way, the longer a person has been homeless, the worse their expected state of health is. Addressing housing exclusion, promoting interpersonal relationships, using a day center, and developing the use and perceived helpfulness of social services stand out as key factors in improving health status. Social policies are usually focused on housing. However, this paper also highlights the relevance of developing interpersonal relationships and using day centers to improve homeless people’s health.

## 1. Introduction

Among the dimensions of housing exclusion, homelessness is a complex issue. The European Typology on Homelessness and Housing Exclusion (ETHOS) [1] covers four forms of housing exclusion: roofless, houseless, insecure and inadequate housing. The first two are associated with homelessness, while the second two are more closely related to residential exclusion in the strict sense [2,3]. Homelessness could be triggered by structural factors like insufficient or inadequate social and health care systems, unaffordable housing, or/and individual factors such as health problems and poverty or mental disorders [4,5]. Over 700,000 homeless people are currently living in Europe [6]. In particular, the homeless population in Spain is estimated to be between 30,250–36,300 [7] with an expected upward trend in the coming years. Some figures to understand this expected trend are, for example, the number of homeless people in a daily emergency and temporary housing situation increased between 2014 and 2015 by 20.5% [8] or 18,001 people who were housed on an average daily basis in care for homeless people in 2018—up 9.5% from 2016 [9]. This increase can also be observed in the counts made in Spain’s largest cities. In Madrid between 2016 and 2018, the number of people living on the street increased by 24% and the number of people in a shelter by 11% [10]; in Barcelona the increase in homelessness was 8% [11]; and in the Basque Country it increased by 58% [12]. Unfortunately, the pandemic caused by COVID-19 has interrupted the counts planned for the year 2020, although an increase in the estimate of the future situation was included in the Comprehensive National Homelessness Strategy 2015–2020” (ENI-PSH in Spanish) [7]. Given this data, the development of new research to better understand the situation of this group in Spain is a key issue.

### 1.1. Personal Variables and Self-Rated Health

The concept of Self-Rated Health (SRH) is based on the subjective assessment of health status, usually on a four- or five-point scale, which can then be used to analyze the risk factors associated with individual health status [13,14]. Previous national [15,16] and international studies have demonstrated the high level of reliability of the SRH measure for measuring objective health [17,18,19] and its use has been suggested as an useful and appropriate indicator in national surveys over decades [20,21]. SRH has become a longstanding tool to measure health characteristics and inequalities [16].

In the case of the homeless population, homelessness triggers health consequences and poses a challenge to health services action and policies [22]. Compared to people with homes, homeless people experience higher use of emergency services [23] and higher prevalence and incidence of medical and psychiatric morbidity and mortality [24]. As Nagy-Borsy et al. (2021) indicated, the material deprivation and the lack of resources constrain their capacity to focus on their health problems and health care needs, and prevent them from health-promoting behavior [5]. It is complex to identify the causes and effects between health and homelessness, so an in-depth analysis of SRH is required to contribute to the improvement of knowledge of the homeless’ experience. Regarding the importance of personal characteristics in SRH, women tend to report more symptoms and greater use of health services, as well as poorer SRH than men [25]. However, this tendency sometimes diverges depending on age, where the likelihood of women reporting poorer SRH is lower for older age groups [26]. For the homeless, recent studies show the same trend with poorer health for women [27,28,29]. Nevertheless, there is no robust evidence on the health of homeless women in Europe [29] and more research would be required [27]. Regarding time being homeless, a diffuse concept, chronicity, has been developed in Europe, which can lead to confusion in the results and make international comparisons difficult [4]. In this sense, this paper considers the time being homeless as a continuous variable measured in years. Previous studies on homelessness show the longer one has been homeless, the poorer the SRH [23] and the higher the probability of mental health difficulties [28]. However, large samples have not been used for the Spanish case.

### 1.2. Interpersonal Relationships, Social Services Support and SRH

Supportive relationships, according to Peplau’s *Theory of Interpersonal Relations* [30,31], are processes between two or more individuals that can be created between clients and service providers, family, and peers [32]. Homeless people are socially isolated, with low levels of social support and social functioning. They lack social and family support or relationships with service providers. On the contrary, supportive relationships promote intrapersonal and interpersonal strengths, resilience, hope, and the forward movement of health [31,33,34]. Therefore, Peplau’s Theory of Interpersonal Relations [30,31] highlights the importance of supportive relationships in achieving health.

It is widely known that homeless people often suffer from serious health problems and that the lack of social resources contributes to their poor health [35]. Social support has been found to have protective effects on physical health outcomes, such as cardiovascular disease and mortality, and mental health outcomes, such as depression and anxiety in general population [36,37,38]. According to youth living in shelters, social support was also a protective factor against poor health [39]. Likewise, a strong positive association has been found between perceived social support and self-rated physical and mental health in the elderly population [40]. Supportive relationships are also closely associated with recovery [41] and higher levels of health and social service utilization among homeless people with serious mental illness [42]. Meeks and Murrell (1994) [43] claimed that both perceived and received forms of social support from informal social network relationships can have greater meaning, significance, and benefits for health and well-being. The reason for this is a greater reciprocity in the perception and receipt of support than the existing through relationships with service providers, who may be more obligated to provide support to clients as part of their job. Thus, social support derived from family, friends, and neighbors may be more meaningful to homeless people and subsequently may have a greater impact on health. However, what does the scientific literature show about social relationship and health in Spain? A recent qualitative study in Spain demonstrated the relevance of the interpersonal relationship on homelessness effectiveness interventions [44]. Twenty interviews with homelessness in Barcelona showed the relevance of the social support of volunteers, professionals, and family in their life and on the potential social impact of the intervention programs. Another study on homeless Spanish women showed how social relationships appear to be a key factor in reducing the feeling of loneliness, and this in turn would have a positive impact on the mental health of homeless Spanish women [45]. However, there are no previous quantitative studies on interpersonal relationships and SRH on homelessness in Spain; even more with a high sample size.

In relation with the social services support, Hwang et al. (2009) [28] found that having used one to two health care services in the last twelve months, and use of three or more health care services in the last twelve months were predictive of poorer physical health status. However, there is no information about these variables in current studies in Spain. Are these associations similar in Spanish homelessness? Likewise, perceived emotional support from social network ties was positively associated with mental health. This shows that perceived access to emotional support within networks was associated with better mental health status. Is the perceived level of help from social services associated with the health of homelessness in Spain? Likewise, currently living in a homeless shelter was positively associated with mental health score. In the same way, Fajardo-Bullón et al. (2019) [13] found that having spent a night in hospital and having gone to the doctor in the last month were all significant risk factors associated with perceived poor health of homelessness. On the other hand, Gasior et al. (2018) [46] analyzed the influence of a network of service providers, perceptions of social supports, and family relations on a homeless youth’s perceptions of recovery from mental illness. They found that services provider networks were not related with perceptions of recovery. However, both, social support and family relations were positively and significantly correlated with perceptions of recovery. That is, social support and family relations were significant predictors in the mental health perceptions of the recovery process of homeless youth. Does the use of mental health and health care services have an influence on the SRH of homelessness in Spain? Is the use of day centers associated with good SRH?

### 1.3. The Present Study

Although there are studies that analyzed the health of homeless people in Spain, they were mainly focused on homeless women with disabilities [47], housing exclusion population (insecure and inadequate housing) [27], or on homelessness (roofless and houseless) [13], according to the 2012 Spanish national survey [48]. Other recent Spanish studies were focused on mortality [49], chronic homelessness in specific cities like Girona [4,50], health of homeless migrants in Valencia [6], and women’s mental health in Madrid [45], but not in SRH. There is a clear need for providing robust data about the adverse health circumstances of homeless people [5] and a need for more country-specific cross-sectional studies in Spain [6]. Specifically, the factors that influence homelessness SRH should be studied with more recent data. Moreover, to the best of our knowledge, this is the first paper that shows evidence of homelessness SRH in the Basque Country. Therefore, in this paper we would like to present new evidence on the association between certain personal characteristics, interpersonal relationships, and the use and perceptions of being helped from social services with SRH in homeless people (roofless and houseless) using a large sample collected by the IV study on the condition of people in a situation of serious residential exclusion in the Basque Country (Spain) carried out in 2018. The relationships between the factors were examined using multinomial logistic regression modelling. Modelling the relationship between explanatory (predictor) and response variables is a fundamental activity encountered in risk analysis [51]. The accounts of these studies suggest that simple linear regression is often used to investigate the relationship between a single predictor variable and a single response (dependent) variable; however, when there are several explanatory variables, multinomial logistic regression is used. However, the response (dependent variable) is often not a numerical value. Instead, the response is simply a designation of one of two possible outcomes (a binary response) [52]. As can be seen from the stated purpose, we will estimate the effects of different factors at the same time on each individual’s self-reported perception of health—not their actual level of health—which falls into different categories. Thus, the analysis seeks to estimate the probability of self-perceiving different states of health by means of a linear combination of the factors considered using multinomial logistic regression. To summarize, the aim of this paper is threefold: (1) To analyze the influence of certain personal variables (gender and time being homeless) on the SRH; (2) To analyze the influence of interpersonal relationships (contacts with family members and spending the day with people/alone) on the SRH; and (3) To analyze the influence of social services used and the perception of being helped from them on the SRH.

## 2. Materials and Methods

This paper used data collected by the IV study on the condition of people in a situation of serious residential exclusion in the Basque Country (Spain) carried out in 2018, where 26 cities participated. The Basque Country is one of the communities most committed to homelessness in Spain, doing biennial studies since 2012. In their continuity, the inter-institutional collaboration protocol for the investigation, monitoring, and analysis of homelessness in the Basque Country of July 2016 has played a fundamental role, in which the local, provincial, and regional administrations agreed to carry out nightly counts every two years. In the Basque Country, the law opens precisely with the explicit recognition of the subjective right of access to the legal occupation of a home by all those who are not in possession of one and do not have the necessary means to achieve it; and defines a homeless person as a person who does not have a physical place of residence, or who lacks housing, or whose dwelling is unsafe or inadequate. Likewise, the Basque Country has developed the Basque Strategy for the Homeless. The Basque strategy is based on an inter-institutional pact in which three departments of the regional executive are involved—Employment and Social Policies; Environment, Territorial Planning and Housing; and Health—as well as the three provincial councils, quite a few cities, and the association of Basque municipalities called Eudel. The strategy proposes fifty concrete guidelines that are grouped into nine priority axes of action. In order to develop the Basque strategy, the progressive construction of a network of public housing for homeless people is proposed.

The field work conducted in this investigation consisted of two different actions or steps. On the one hand, the night street count carried out on the night of October 18 to 19; and on the other hand, the interviews carried out in the different services the day after. A total of 920 volunteers participated in the night count, divided into different groups. Each group went out between 10 pm and 12 am to look for homeless people in one of the specific areas into which the cities had been divided as well. When they found a homeless person, they asked them about their willingness to participate in the study by answering a questionnaire. The next day, the managers and employees of the services used by the homeless used the same questionnaire to ask the users about their situation and their characteristics. Therefore, the interviews in the services were develop by employees and the interviews in the night street count were developed by employees and volunteers, who were trained before the night street count to know how to conduct the interview. More information can be found on the web of the IV study on the condition of people in a situation of serious residential exclusion in the Basque Country (Spain) [12].

### 2.1. Participants

**Figure 1:**
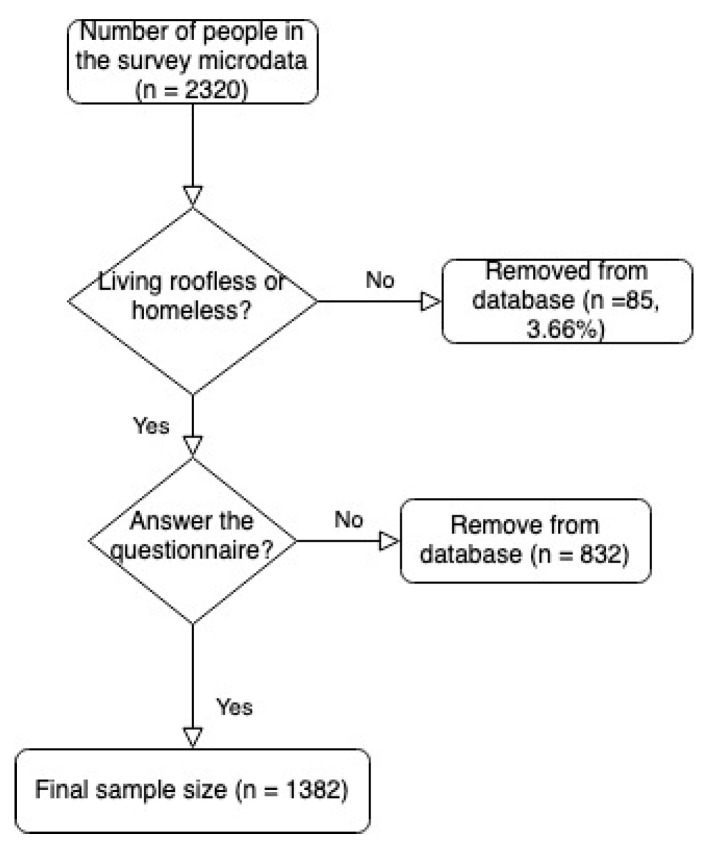
Flow diagram of survey participants.

The total number of people who were identified in the original study were 2320 individuals (see Figure 1). However, for the purposes of this research, two decisions were made. In the first step, only people in the first two ETHOS categories that define homelessness (roofless and houseless) were selected (*n* = 2235). In the second step, people who answered the entire survey were considered. That is, from the 2235 persons who were in roofless and houseless categories, only 1382 decided to participate in the survey, so 832 people did not want to answer the entire survey and only general information on very few variables about them was collected. For this reason, there was no missing data because the 1382 people who decided to be fully interviewed answered all the questions of the survey. In short, the final sample selected for this paper consisted of 1382 homeless, where 1046 were male (75.69%) and 336 were female (24.31%).

### 2.2. Instrument

Although the questionnaire that the participants involved in this study completed was longer, five items were selected for the purposes of this study:

1. How long have you been living without accommodation that you can consider your home? We refer to the time that you have been homeless, that is, sleeping on the street, in a shelter or from one pension to another (time being homeless). Participants had to write down how many years and months they had been in that situation; if it took less than one year, they had to point the number of months.
2. Family and social relationships. This section had two items: (1) In general, do you have a family member with whom you have contact from time to time, either in person and/or through the phone, mail, or internet? The response options were two, Yes or No; and (2) Do you usually spend most of the day alone or do you often spend time together with other people? The response options were also two, I spend most of the day alone, or I spend most of the day with other people.
3. Use of services. The question was, “In the last six months of the services or benefits mentioned below, could you say which ones you have used?” The response options were day center, health center or hospital, and mental health center. Each question had two response options: Yes or No.
4. Perception of being helped from social services. The question asked, “Please tell me considering your experience if social services have helped you” and had four options: None, Low, Sufficient, or High.
5. Health perception. The question was, “In general, how would you say your health is?”, with five answer options: Very good, Good, Regular, Bad, or Very bad.

### 2.3. Variables

The variable under study in this analysis is the state of health perceived by homeless people (Dependant variable, DV). This categorical variable has been constructed from the responses collected in the survey on this issue, ordered from very bad to very good in five categories. However, to avoid problems of low sample size in some of these original categories, the extremes have been grouped together. Therefore, the self-rated health variable used in the study comprises of three categories: (1) good—grouping the original categories “very good” and “good”—(2) regular, and (3) bad—containing the original categories “very bad” and “bad”, with “good” as the base category. The explanatory variables (Independent variables, IVs) can be grouped into three dimensions: personal variables (gender and time being homeless), interpersonal relationships (relationships with family and spending the day alone/with people), and institutional support (use of health centers, use of mental health centers, use of day centers and perception of the level of help provided by social services). Except time living homeless—the only metric variable measured in years—and the perceived level of help provided by social services (with four categories: None, Low, Sufficient and High), the rest of the variables are binary and take the value 1, respectively, if the person is a woman, has contact with family, spends the day alone, has gone to a health center, has gone to a mental health center, or has gone to a day center. Table 1 reports the descriptive analysis for these variables.

### 2.4. Statistical Analysis

The impact of the explanatory variables on the dependent variable were measured using a multinomial adjusted logit model. Thus, we estimate the probability of reporting a fair or poor health status versus the base category, good health status. Specifically, this methodology expresses the ratio of the probability of one of the alternative states and the probability of the base state as a function of the values of the explanatory variables and their respective coefficients. The estimates of the beta coefficients presented in Table 2 will involve an increase in the probability of the alternative states—worse health—if they are positive and a decrease in the same probabilities—better health—if they are negative. In addition, the relative risk ratios, whose interpretation is based on being greater (increases the relative probability) or less (decreases) than one, are reported. The analysis was performed by using the statistical software Stata v.15 for OSX.

As shown in Section 1.2, the impact on SRH status of two sets of factors will be estimated: those related to interpersonal relationships and those related to the social devices available to the homeless. Together with these, different control variables should be considered to capture the influence of specific characteristics of individuals.

An iterative process of estimation of several multinomial regression models is carried out starting from the saturated model, which includes not only the variables under study, but also other possible explanatory variables such as attendance at soup kitchens or control variables such as level of education (This successive elimination process is performed to avoid misspecification due to the large number of variables available in the study, whose joint estimation affects the overall significance as well as that of each variable. The complete list of variables in the survey can be obtained from the authors upon request.). These are successively eliminated according to their lack of significance and the respective reduced models are analyzed. Total elimination of all the non-significant variables of the saturated model is not carried out to avoid a specification error. Once the non-significance of the variables not included in the theoretical model has been verified, the likelihood of the estimated model is analyzed, and the robustness of the results is checked by replicating the estimation with other functional forms such as binary logit, ordinal logit, or multinomial probit.

As opposed to the binary logit that estimates the probability or risk of being in a category using as a reference not being in it, independently of the possible states outside the category of interest, the multinomial logit model estimates, taking one of the possible categories as a reference, the probability or risk of being in the rest of the possible states. This avoids the misidentification of the effects caused by factors that may be significant for one of the categories of the dependent variable and not for another, or, both being significant, have different impacts.

In addition, the multivariate nature of the estimated models means that the effects measured in the beta coefficients or relative risk ratios of each variable are estimates adjusted to the values of the other variables considered, avoiding the possible distorting effect caused by confounding variables. Finally, the constant term of the model allows adjusting the estimates with respect to the variables omitted in the final formulation of the model. After selecting the best covariates and testing the goodness of fit of the model, different model specifications are tested, such as two binary logits (bad SRH vs. non-bad SRH and good SRH vs. non-good SRH), an ordinal logit and a multinomial probit model (see, respectively, Table A1, Table A2, Table A3 and Table A4 in Appendix A), whose respective results are similar to those shown in Table 3 and do not improve the information provided by the multinomial logit model. Consequently, one can be confident in the robustness of the estimation made after this sensitivity analysis.

## 3. Results

Table 1 reports the main characteristics of the survey participants. The question of gender stands out significantly as the majority are men. With regards the time they have been living homeless, the figure is very striking, because they have been experiencing this situation for an average of just over four years. In addition, the information on social relationships describes most individuals with occasional contacts with family, while the percentage of people spending the day alone does not reach 30%. In other words, the social support of these individuals appears to be, apparently, good. On the other hand, data on the use of the different services provide very disparate information. While almost all say they have used day centers, three-quarters say they have gone to the health center and only about one-third have gone to the mental health center. Finally, regarding the perceived level of assistance from social services, the responses show a positive evaluation. More than 60% of the participants stated that the level of assistance was sufficient or high. In relation to the dependent variable, almost 60% of individuals report a good subjective health status, with a low incidence of poor health status. This outcome supports choosing “good SRH” as the baseline category.

After describing the frequencies and means of the independent or explanatory variables together with SRH status, the explained variable, and before presenting the final model, the unadjusted relative risk ratios are shown in Table 2. This data can be compared with those reported in Table 3, already adjusted or controlled for the rest of the variables (even the omitted variables are controlled for by the coefficient of the constant term.). Its interpretation is easy and widely known. For example, the value of 1.61 for the variable spending the day alone for the “bad” category of SRH status means that spending the day alone makes a bad SRH 1.61 times more likely than a good SRH. The significance of these ratios is outstanding for most of the variables, and above all, for the SRH status category, which reflects a poor perception. The variables that seem to be most related to poorer perceived health are those associated with health care, such as attendance at a health center or mental health service. On the other hand, the help from social services perceived by individuals increases the probability of reporting a good SRH compared to that of reporting a bad one.

The SRH as a dependent variable, as well as the relationship between this variable and the factors described in the previous section, are estimated by using a multinomial logistic regression model. The outcome is the best possible model shown in Table 3 with a L2 = 128.59 (0.0000), meaning that the proposed model as a whole fits significantly better than an empty model. To make the results easier to understand, both adjusted estimates—fair vs. good and poor vs. good—are presented in two columns with the confidence intervals of the coefficient estimates in brackets. In the perceived level of social services assistance, it is necessary to eliminate one of the categories to avoid multicollinearity. As can be seen in Table 2, the “nothing” category is taken as the reference in this case. In general, the coefficients (and relative risk ratios) show the expected signs and values according to theory, although the level of significance is much higher in the “bad” vs. “good” case than in the “fair” vs. “good” case. Controlling the effects of other variables improves the statistical significance of some of the non-significant factors in Table 2. In addition, the relative ratios of most of the explanatory variables are lower once the estimates are adjusted. Besides, a sensitivity analysis was performed by replicating the estimation with different specifications, the results of which are shown in Appendix A. Although there are slight differences in the significance and magnitude of the effects, the results are similar to those obtained in the logistic multinomial regression shown in Table 3. The probabilities of reporting a bad or good SRH were estimated using two binary logit models, the probability of reporting a given SRH considering this variable as ordinal and, finally, a different functional form, probit instead of logit, was used for the non-binary categorical dependent variable. Given the similarity discussed above, we believe it is possible to assume the validity of the model used.

The results in Table 3 show the different profiles, both in significance and magnitude, presented by the two SRH categories compared to the SRH status “good”, which facilitates the analysis necessary to test whether reality supports the proposed theoretical hypotheses.

## 4. Discussion

### 4.1. Personal Variables and SRH

The first goal of this study was to analyze the influence of some personal variables (gender and time being homeless) on the SRH. The two variables included in this dimension present a radically different situation. On the one hand, gender not only has different signs in each estimation, but also is not significant for any of the alternative states considered; this result does not imply that the gender of the homeless does not matter; indeed, unadjusted estimates indicate that it increases the relative risk of worse perceived health status, but that, unfortunately, there is insufficient evidence to support this impact. Meanwhile, time living homeless is highly significant and shows the expected signs in both cases. A one-unit increase in this variable—i.e., one more year—is associated with an increase in the relative log odds of reporting any of the alternative SRH vs. “good”.

Results have shown that gender is not significantly related with SRH and this is consistent with some previous studies [26], where it was found that men and women’s concurrent SRH validity was similar for comparable health factors. However, time being homeless has shown a noteworthy influence on SRH. People who have spent more time being homeless perceived themselves with poorer health than people who have spent less time being homeless. This result shows the expected relationships between time being homeless and SRH, and is in line with some previous studies [23,53]. Long term homelessness is especially associated with poor health [54,55]. Besides, it contributes to chronic illnesses and obstructs the interactions with health care providers [56]. Prolonged outdoor exposure, as well as street culture and substance use, can impact adversely on health and life expectancy [13,57]. On the contrary, when homeless people move into permanent supportive housing and leave homelessness, the SRH improves significantly in the first three months after leaving homelessness [58].

### 4.2. Interpersonal Relationships and SRH

The second aim of this study was to analyze the influence of some interpersonal relationships (contacts with family members or spending the day with people/alone) on the SRH. Again, the effects of these variables correspond to what was expected a priori. It is assumed that having contact with one’s family, albeit occasional, when one is homeless helps to have a better state of health due to this family support. Likewise, it is also expected that someone spending the day alone will have a worse state of health than if they live accompanied. Regarding the coefficients of the first variable, both are negative, although it is not significant at 5% in the case of regular SRH. For the bad SRH, it means that the probability of presenting it is lower than that of the good SRH. In other words, we have statistical support to say that occasional contacts with the family improve perceived health status. People who have a family member with whom they have a relationship with from time to time show a better perception of their health than people without family contacts. This result is consistent with Peplau’s Theory of Interpersonal Relations [30,31] that highlights the importance of supportive relationships in achieving health. This result is also consistent with some previous studies [46], which found that family relations were significant predictors in mental health perceptions of the recovery process of homeless youth. Although homeless people have sometimes volatile relationships with family members, these results confirm that family relationships are important for health [33,34]. Therefore, service providers should consider that rebuilding familial relations could improve homeless people’s health. A study focused on rebuilding familial relationships [59] found that 14.5% of analyzed people reconciled a damaged relationship with a family member and 17% moved back home after individual and family counseling. Winland et al. (2011) expose that specific family members can be important in securing an environment of supportive relationships required in the process of mental health recovery [59]. Likewise, Kurtz et al. (2000) [39] found that, despite the volatility in relationships with specific family members, others (siblings, grandparents, aunts, and uncles) can be sources of encouragement, and support during critical life moments. Therefore, some family relations could be utilized to provide support. On the other hand, our results have found that spending the day with other people has also a positive influence in the perception of health. This result follows our expectations and is in line with previous studies, which claim that social support has protective effects on physical health outcomes [36,37,38,39,40,41]. Besides, positive health effects of elements of social capital derived specifically from relationships with nonmarginalized individuals have been found in studies with homeless populations [60,61]. Both outcomes found in our study related with family and social relationships are consistent with Peplau’s Theory of Interpersonal Relations [30,31], which states the importance of supportive relationships in achieving health. Therefore, securing these supports throughout intervention programs may be beneficial.

### 4.3. Social Services Support and SRH

Finally, our third goal was to analyze the influence of using some social services and the perception of being helped from them on the SRH. In this block, it is possible to distinguish between health-related factors and other support. In the first group, we find the use of health centers or mental health centers. Both are significant for both regular and bad health status and present the expected, positive sign. They can be interpreted as a higher relative risk of worse health status if the individual reports having used these services. Moreover, as would be expected, the impact on the probability will be greater if the SRH is bad. Regarding other institutional supports, they share one characteristic: no significant impact for regular health status, while the opposite is the case for poor health status. Specifically, the use of day-care centers shows a markedly significant reducing effect on the relative risk of bad SRH. At the same time, the support of social services stands out as a key issue in reducing the risk of having a bad SRH, as can be deduced from the negative signs of the levels “sufficient” and “high”.

The results show that the use of health or mental centers are related with poorer health, as expected. That is, if homeless people use both centers, it means that their health is not as good as they would like. These results are in line with those studies that found that having used one to two health care services in the last twelve months and use of three or more health care services in the last twelve months were predictors of poorer physical health status [28] or other papers that found that having spent a night in the hospital and having gone to the doctor in the last month were all significant risk factors associated with perceived poor health of homeless people [13]. On the other hand, the use of day centers has a striking positive relationship (the highest in our study) with the health perception. That is, people who use day centers, where they have the possibility to socialize, meet people to talk or play something with, has a positive effect of their perceptions of health. Also, people who perceive higher levels of support from social services have a better perception of their health. These results are consistent with the finding of social support related to higher levels of health and social service utilization among homeless persons in other studies [42,46]. In a recent systematic review of homeless persons’ experiences of health- and social care, being cared for and respected in the professional relationship were described as supportive, while experiences of alienation, discrimination, disrespect, and stigmatisation were experienced as unhelpful [62]. These findings highlight the need for more services encouraging the integration of homeless people into social networks. For example, McCay et al. (2011) [63] examined the impact of relationship-based interventions for homeless youth in Toronto. Participants experienced improvements in social connectedness and decreased hopelessness after six sessions with a clinician. On the contrary, those who did not participate in the intervention experienced increased levels of mental health symptoms. These findings suggest that supportive relationships with service providers may strengthen homeless social relationships and mitigate overwhelming hopelessness. According to persons experiencing homelessness, as Omerov et al. described (2020) [62], possible strategies to reduce barriers for good health and wellbeing include societal support to accommodate basic human needs, supportive relations with professionals from health and social care, and special programs that integrate health and social care, including flexible, drop-in services.

### 4.4. Limitations

It is important to pinpoint some possible limitations of the research paper. Although we argued for the validity of SRH, the use of self-reporting for general health and the remainder of the associated variables analyzed may be considered a limitation themselves. Data are self-reported and subject to recall and social desirability bias. However, this is a common methodology used in big national surveys in Spain [16,64] and traditionally recommended to be used internationally [18,20]. Another limitation is that some of the data collection occurred when people attended services. However, this methodology is used and recommended by the Spanish National Statistics Institute on Homelessness surveys. The number of women represent 24.7% of the sample. It is not a balanced proportion in regard to gender. However, the European Parliament recognize that homelessness is a “gendered phenomenon” in Europe, where 75% of homeless people are men [65], similar to our participants. Women usually represent a minority among the homeless population surveyed, rarely accounting for more than 20–30% of the total [66]. In these terms, in the present study there is a lack of gendered experiences, as women who are homeless have reduced outcomes compared to men and the sample sizes are not large enough to allow one to infer significant differences. It would be interesting to develop new studies with a larger sample size, and consequently, to analyze the possible existence of gender inequality in the situation of homeless people, including data representing only a minority of women. On the other hand, social support is a multidimensional concept and only a couple of items have been used in this study to measure family and social support or relationships. Therefore, future research should measure social support more profoundly, by looking at the size of social networks, as well as received and perceived emotional, financial, and instrumental social support, etc. Besides, as Hwang et al. (2009) [28] claimed, longitudinal research that explores the influence of social relationships on the health of homeless populations is needed to determine if a high demand for social support among these marginalized individuals may erode their relationships and subsequently lead to poorer mental and physical health and victimization over time. Finally, race is not included in the survey and the authors could not adjust the analyses for this variable.

Although this research has some limitations, it can be reliably assumed that its results contribute to reinforce the significant effects that social or interpersonal relationships have on the health perception of homeless people in Spain. Usually, interventions with the homeless are focused on providing basic needs, like food, accommodation, or a shower, but family and social relationships are forgotten. The findings reported in this study highlight the importance of family, social and social service providers influence on SRH. Therefore, interventions with the homeless should consider these results and try to rebuild family and social relationships. Our results support how the development of social networks improve the health of the homeless in the Basque Country. In this sense, Rolfe et al. (2020) [55] demonstrated that housing can also improve health and well-being by improving relationships. When temporary accommodation becomes a home, this improved situation can reduce stress and create a sense of status. Besides, in a good-quality neighborhood, environments and networks can increase opportunities for socialization, improving health and well-being. According to our findings and previous theories [54,55], having a roof over one’s head while promoting the use of day centers or improving family relationships will result in even more positive health effects.

The social service’s perceived help should be also highlighted. Social policies should understand that if the help received by the homeless is, at least, sufficient, the positive effect on health will be higher and it may pose a better relation between health services and homeless people. Housing can also support better health, improving access to health care services and the interaction with the health care providers at the same time [57,67]. Finally, our results show how mental health and healthcare services are used by the homeless when they perceive poor SRH. Promoting more preventive policies with periodic health reviews could be a good initiative to hamper diseases and chronicity. Nevertheless, more research is needed to consider new factors that could improve the health of homeless people and generate new health and social policies for the homeless population.

## 5. Conclusions

In summary, the results of this research show that: (1) Time spent homeless is highly significant; the longer a person has been homeless, the worse their expected self-perceived health is; (2) Interpersonal relationships are related to health perception; having contact with one’s family and spending the day with somebody helps to increase the perception of good self-rated health; (3) The use of health centers and mental health services is associated with poor health; and, (4) Using a day center and the perception being helped by social services are related to better health perception. Further research is needed to expand the model and study the impact of various prevention policies and initiatives related to factors contributing to the health of the homeless population.

## Figures and Tables

**Table 1:** Descriptive statistics ^1^.

**Variable (IV)**	**N (number of observations)**	**Percentage**
Gender (female)	336	24.31%
Family relationships ^a^	1170	84.66%
Spending the day alone ^a^	392	28.36%
Use of health care services ^a^	1036	74.96%
Use of mental health services ^a^	403	29.16%
Use of day centers ^a^	1307	94.57%
Perceived level of help from social services		
None	141	10.20%
Low	352	25.50%
Sufficient	475	34.39%
High	413	29.91%
**Variable** **(IV)**	**Mean (years)**	**S.D.**
Time being homeless	4.3602	5.9966
**Explained variable (DV)**	**N (number of observations)**	**Percentage**
Good health	796	56.70%
Regular health	409	29.59%
Bad health	177	12.81%

^1^ Source: IV study on the condition of people in a situation of serious residential exclusion in the Basque Country 2018. ^a^ the answer is yes, a confirmation of developing family relationships, spending the day alone, using health care and using days centers.

**Table 2:** Unadjusted estimates (relative risk ratios) of the impact of some variables on the self-perceived health status.

Variables (IV)	“Regular” vs. “Good” RR ^b^ [IC (95%)]	“Bad” vs. “Good” RR ^b^ [IC (95%)]
Gender (female)	1.04 [0.79, 1.37]	1.16 [0.80, 1.68]
Time being homeless ^a^	1.02 ** [1.01, 1.02]	1.01 ** [1.00, 1.03]
Family relationships	0.85 [0.61, 1.19]	0.56 ** [0.37, 0.84]
Spending the day alone	1.39 ** [1.07, 1.81]	1.61 ** [1.13, 2.27]
Use of mental health services	2.05 ** [1.58, 2.66]	2.57 ** [1.83, 3.62]
Use of health care services	2.29 ** [1.70, 3.09]	2.97 ** [1.88, 4.68]
Use of day center	1.40 [0.78, 2.52]	0.57 [0.32, 1.05]
Perceived help by social services		
Low	1.28 [0.80, 2.06]	0.60 [0.34, 1.05]
Sufficient	1.12 [0.71, 1.78]	0.52 ** [0.31, 0.89]
High	1.03 [0.64, 1.66]	0.52 ** [0.30, 0.89]

Source: Authors’ elaboration from Stata 15. **: 5% significance. ^a^ This is a metric variable where the unit is number of years. ^b^ RR = Risk Ratio.

**Table 3:** Impact of some variables on the self-perceived health status (“good” status fixed as base outcome).

Categories	Variables (IV)	“Regular” vs. “Good”		“Bad” vs. “Good”	
		β ^a^	R.R. ^b^	β	R.R.
Personal variables	Gender (female)	−0.06 [−0.35, 0.24]	0.94	0.07 [−0.32, 0.46]	1.07
	Time being homeless ^c^	0.014 ** [0.00, 0.02]	1.01 **	0.01 ** [0.00, 0.02]	1.01 **
Interpersonal relationships	Family relationships	−0.12 [−0.47, 0.23]	0.89	−0.45 ** [−0.88, −0.01]	0.64 **
	Spending the day alone	0.37 ** [0.10, 0.65]	1.45 **	0.45 ** [0.08, 0.82]	1.57 **
Use of social services and perceived help	Use of mental health services	0.55 ** [0.27, 0.83]	1.73 **	0.79 ** [0.43, 1.16]	2.21 **
	Use of health care services	0.78 ** [0.46, 1.11]	2.19 **	1.30 ** [0.77, 1.83]	3.67 **
	Use of day center	−0.11 [−0.75, 0.53]	0.89	−1.10 ** [−1.82, −0.39]	0.33 **
	Perceived help by social services				
	Low	0.23 [−0.27, 0.73]	1.26	−0.43 [−1.02, 0.15]	0.64
	Sufficient	0.00 [−0.49, 0.49]	0.99	−0.68 ** [−1.25, −0.09]	0.51 **
	High	−0.06 [−0.56, 0.43]	0.99	−0.63 ** [−1.22,−0.04]	0.53 **
	Constant	−1.45 ** [−2.18,−0.72]	0.25 **	−1.12 ** [−1.90,−0.35]	0.30 **
	L2	128.59 (0.0000)			
	Sample size	1382			

^a^ B coefficients. ^b^ Relative Risk Ratio. ^c^ This is a metric variable where the unit is number of years. Source: Authors’ elaboration from Stata 15. **: 5% significance.

## Data Availability

Data can be requested in the Statistics Basque Country Institute https://www.eustat.eus/estadisticas/tema_219/opt_0/ti_personas-sin-hogar/temas.html, accessed on 31 August 2021, or the Center of Documentation and Studies (https://www.siis.net/es/contacto/, accessed on 31 August 2021). The dataset used analyzed during the current study is available from the corresponding author on reasonable request.

## Appendix A

**Table A1:** Impact of some variables on a bad self-perceived health status (binary logit).

Categories	Variables	Bad vs. Non-Bad	
		β ^a^	O.R. ^b^
Personal variables	Gender (female)	0.89 [−0.28, 0.46]	1.09
	Time being homeless ^c^	0.004 [−0.005, 0.15]	1.004
Interpersonal relationships	Family relationships	−0.40 ** [−0.81, −0.009]	0.67
	Spending the day alone	0.30 [−0.45, 0.65]	1.35
Use of social services and perceived help	Use of mental health services	0.57 ** [0.23, 0.92]	1.77 **
	Use of health care services	1.05 ** [0.46, 1.58]	2.86 **
	Use of day center	−1.06 ** [−1.74, −0.38]	0.35 **
	Perceived help by social services		
	Low	−0.52 [−1.08, 0.32]	0.59
	Sufficient	−0.67 ** [−1.22, 0.12]	0.51 **
	High	−0.60 ** [−1.16, 0.04]	0.99 **
	Constant	−1.36 ** [−2.23,−0.54]	0.25 **
	L2	56.40 (0.0000)	
	Sample size	1382	

^a^ B coefficients. ^b^ Odds Ratio. ^c^ This is a metric variable where the unit is number of years. Source: Authors’ elaboration from Stata 15. **: 5% significance.

**Table A2:** Impact of some variables on a good self-perceived health status (binary logit).

Categories	Variables	Good vs. Non-Good	
		β ^a^	O.R. ^b^
Personal variables	Gender (female)	0.02 [−0.24, 0.28]	1.02
	Time being homeless ^c^	−0.01 ** [−0.02, −0.004]	0.98 **
Interpersonal relationships	Family relationships	0.22 [−0.09, 0.53]	1.25
	Spending the day alone	−0.39 ** [−0.64, −0.15]	0.67 **
Use of social services and perceived help	Use of mental health services	−0.62 ** [−0.87, −0.37]	0.53 **
	Use of health care services	−0.92 ** [−1.21, −0.62]	0.40 **
	Use of day center	0.46 [−0.07, 0.99]	1.58
	Perceived help by social services		
	Low	0.001 [−0.43, 0.43]	1.001
	Sufficient	0.24 [−0.19, 0.66]	1.27
	High	0.27 [−0.16, 0.70]	1.31
	Constant	0.60 [−0.06, 1.26]	1.82
	L2	108.91 (0.0000)	
	Sample size	1382	

^a^ B coefficients. ^b^ Odds Ratio. ^c^ This is a metric variable where the unit is number of years. Source: Authors’ elaboration from Stata 15. **: 5% significance.

**Table A3:** Impact of some variables on the self-perceived health status (ordinal logit).

Categories	Variables	β ^a^	O.R. ^b^
Personal variables	Gender (female)	0.05 [−0.24, 0.28]	1.004
	Time being homeless ^c^	0.01 ** [−0.02, −0.004]	1.002 **
Interpersonal Relationships	Family relationships	0.25 [−0.09, 0.53]	0.78
	Spending the day alone	0.37 ** [−0.64, −0.15]	1.44 **
Use of social services and perceived help	Use of mental health services	0.63 ** [−0.87, −0.37]	1.87 **
	Use of health care services	0.93 ** [−1.21, −0.62]	2.53 **
	Use of day center	−0.62 ** [−0.07, 0.99]	0.54 **
	Perceived help by social services		
	Low	−0.02 [−0.43, 0.43]	0.85
	Sufficient	−0.37 [−0.19, 0.66]	0.69
	High	−0.40 [−0.16, 0.70]	0.68
	L2	116.27 (0.0000)	
	Sample size	1382	

^a^ B coefficients. ^b^ Odds Ratio. ^c^ This is a metric variable where the unit is number of years. Source: Authors’ elaboration from Stata 15. **: 5% significance.

**Table A4:** Impact of some variables on the self-perceived health status (multinomial probit, good SRH as base category).

Categories	Variables	“Regular” vs. “Good”	“Bad” vs. “Good”
		β ^a^	β ^a^
Personal variables	Gender (female)	−0.84 [−0.28, 0.19]	0.39 [−0.24, 0.32]
	Time being homeless ^b^	0.01 ** [0.004, 0.02]	0.008 [−0.004, 0.02]
Interpersonal Relationships	Family relationships	−0.10 [−0.38, 0.18]	−0.34 ** [−0.66, −0.03]
	Spending the day alone	0.31 [0.08, 0.53]	0.34 ** [0.08, 0.61]
Use of social services and perceived help	Use of mental health services	0.46 ** [0.23, 0.68]	0.59 ** [0.32, 0.85]
	Use of health care services	0.65 ** [0.39, 0.91]	0.95 ** [0.60, 1.30]
	Use of day center	−0.13 [−0.63, 0.37]	−0.77 ** [−1.31, 0.24]
	Perceived help by social services		
	Low	0.16 [−0.23, 0.56]	−0.29 [−0.73, 0.15]
	Sufficient	−0.02 [−0.41, 0.36]	−0.46 ** [−0.91, 0.05]
	High	−0.07 [−0.47, 0.32]	−0.44 ** [−0.88, 0.002]
	Constant	−1.11 ** [−1.73,−0.49]	−0.94 ** [−1.60,−0.28]
	Log likelihood	−1236.8535 (0.0000)	
	Sample size	1382	

^a^ B coefficients. ^b^ This is a metric variable where the unit is number of years. Source: Authors’ elaboration from Stata 15. **: 5% significance.
